# Immunohistochemical characterization and functional identification of mammary gland telocytes in the self-assembly of reconstituted breast cancer tissue *in vitro*

**DOI:** 10.1111/j.1582-4934.2012.01646.x

**Published:** 2012-12-04

**Authors:** Yongchao Mou, Yan Wang, Junjie Li, Shuanghong Lü, Cuimi Duan, Zhiyan Du, Guili Yang, Weizhen Chen, Siyang Zhao, Jin Zhou, Changyong Wang

**Affiliations:** a Department of Advanced Interdisciplinary Studies, Institute of Basic Medical Sciences and Tissue Engineering Research Center, Academy of Military Medical Sciences27 Taiping Rd, Beijing, China; b Department of Life Science and Engineering, Harbin Institute of TechnologyHarbin, China; c Laboratory of Oncology, Affiliated Hospital of Academy of Military Medical SciencesBeijing, China

**Keywords:** Telocytes, reconstituted breast cancer tissue, collagen/Matrigel scaffolds, stromal cells, self-assembly

## Abstract

Telocyte (TC) as a special stromal cell exists in mammary gland and might play an important role in the balance of epithelium-stroma of mammary gland. Considering that different types of breast interstitial cells influence the development and progression of breast cancer, TCs may have its distinct role in this process. We here studied the roles of TCs in the self-assembly of reconstituted breast cancer tissue. We co-cultured primary isolated TCs and other breast stromal cells with breast cancer EMT-6 cells in collagen/Matrigel scaffolds to reconstitute breast cancer tissue *in vitro*. Using histology methods, we investigated the immunohistochemical characteristics and potential functions of TCs in reconstituted breast cancer tissue. TCs in primary mammary gland stromal cells with long and thin overlapping cytoplasmic processes, expressed c-kit/CD117, CD34 and vimentin in reconstitute breast cancer tissue. The transmission electron microscopy showed that the telocyte-like cells closely communicated with breast cancer cells as well as other stromal cells, and might serve as a bridge that directly linked the adjacent cells through membrane-to-membrane contact. Compared with cancer tissue sheets of EMT-6 alone, PCNA proliferation index analysis and TUNEL assay showed that TCs and other breast stromal cells facilitated the formation of typical nest structure, promoted the proliferation of breast cancer cells, and inhibited their apoptosis. In conclusion, we successfully reconstituted breast cancer tissue *in vitro*, and it seems to be attractive that TCs had potential functions in self-assembly of EMT-6/stromal cells reconstituted breast cancer tissue.

## Introduction

Mammary gland telocytes (TCs) were identified as special populations in stromal cells in mammary gland by Popescu and his co-workers [1, 2]. During the last few years, the presence of TCs has been documented in different organs and tissues, such as mammary gland [3, 4], epicardium [5], myocardium [6], endocardium [7], lungs and pulmonary veins [8, 9], male genital organs [10], uterus as well as fallopian tube [11], placenta [12], gallbladder [13], oesophagus [14], pancreas [15], meninges, choroid plexus [16] and skin [17]. TCs shared 2–3 long (20–80 μm), thin, moniliform prolongations suddenly emerging from a small, and oval cell body [1]. Researchers proposed that TCs might have functions in the intercellular communication and pace making [1, 18]. In experimental acute myocardial infarction, TCs were demonstrated to be in close spatial relationships with endothelial tubes through direct physical interaction or indirectly by paracrine secretion including VEGF and NOS2 within the ‘angiogenic zones’, which suggests the participation of TCs in neo-angiogenesis during the late stage of myocardial infarction [19]. Recently, Ardeleanu *et al*. studied the relationship between TCs and PEComas and GISTs. They propose TCs as the common cell origin of both PEComas and GISTs [20]. This suggests that the participation TCs might be associated with tumour genesis and biology.

Breast cancer is a most common malignant disease for women, while the underlying etiological mechanisms have not been clarified [21]. In development and progression of breast cancer, stromal cells as an important component of stroma contribute to tumour microenvironment, fostering cancer cells. Orimo *et al*. demonstrated that fibroblasts can promote tumour growth and angiogenesis in invasive breast cancer [22]. It was shown that endothelial cells affect the oxygen-sensing pathways, which lead to inhibit the invasion and metastasis of cancer cells [23]. Adipocytes can secrete adipokines including leptin, ApN and HGF, which have angiogenic properties and promote invasion [24, 25]. The intercellular communications between breast cancer cells and stromal cells are essential for the structural assembly as well as malignant phenotype of breast cancer [26–28]. Until now, there has been no evidence showing that TCs participate the self-assembly of breast cancer.

Traditionally, the most common approaches to explore the functions of stromal cells to breast cancer are through 2D cell culture, animal models or tissues originated from patient. However, 2D cell culture was confined by the shortage of three-dimensional (3D) tumour microenvironment *in vivo*. Animal models were limited by their sophisticated physiological conditions. Moreover, tissues from patient were confronted with the ethical problems. In recent years, the engineered breast cancer tissues *in vitro* reconstituted by different materials and different seeding cells have been used to study mechanisms of migration, metastasis and angiogenesis of breast cancer. Baker *et al*. studied the mutual effect among cell motility and 3D collagen properties including stiffness and architecture in progression of a mammary epithelial cell (MEC) cancer [29]. In Matrigel 3D culture system, researchers revealed that TGFβ regulated Par6 signalling directly to influence the metastasis of cancer cells [30]. Fischbach *et al*. found that IL-8 secretion was affected by integrin engagement and the angiogenic signal was regulated by tumour microenvironment through comparing the angiogenic properties in 2D and 3D alginate hydrogel [31]. Therefore, the tumour tissue reconstitution *in vitro* was an ideal tool for studying the behaviours of tumour cells under specific tumour microenvironment.

Stromal cells as important components of tumour microenvironment have been studied *via* 3D culture. Fibroblasts can promote the invasion of tumour cells in 3D Matrigel through upregulating MMP-2 activity and metastasis promoting S100A4 protein [32], and potentiating cancer cells proliferation in Matrigel co-culture system [33]. Adipocytes, instead of preadipocytes, could enhance the growth of tumour cells in 3D collagen culture, and the expression of E-cadherin was not influenced by both adipocytes and preadipocytes [34]. Endothelial cells induced epithelial to mesenchymal transition (EMT) of breast cancer cells through manipulating the expression of E-cadhein to N-cadherin, and promoted the capability of migration, especially making cancer cells acquire cancer stem-cell character [35].

Although there were many studies reported on the function of different kinds of stromal cells to breast cancer, the relationship of specific interstitial cell, TCs with breast cancer has not been investigated. In this work, we aimed to characterize TCs in EMT-6/stromal cells reconstituted breast cancer tissue, to try to assess their potential function in self-assembly of reconstituted breast cancer tissue *in vitro*. This study would provide evidence of TCs for influencing the typical nest structure assembly in breast cancer and the biological properties of cancer cells.

## Materials and methods

### Chemicals and cell culture reagents

HEPES was purchased from Sigma-Aldrich (St. Louis, MO, USA). DMEM, RMPI Medium 1640 and DMEM/F12 were purchased from Gibco/Invitrogen (Life Technologies Corporation, NY, USA). Foetal bovine serum (FBS) was purchased from Sigma-Aldrich. Matrigel™ and rat tail type I collagen were purchased from BD Biosciences (Franklin Lakes, NJ, USA). Methylene blue solution was purchased from Merck (Merck KGaA, Darmstadt, Germany). Phalloidin-FITC was purchased from Sigma-Aldrich.

### Cell maintenance

The breast cancer cell line EMT-6 was derived from a BALB/c mouse after implantation of a hyperplastic mammary alveolar nodule, and it was purchased from the American type culture collection (Rockville, MD, USA). EMT-6 was cultured in RMPI medium 1640 (Sigma Chemical, St. Louis, MO, USA) containing 10% FBS (Sigma Chemical). Primary isolated stromal cells were cultured in the medium of DEMEM/F12 (Gibco) containing 10% FBS. Reconstituted breast cancer tissues, by mixing EMT-6 with stromal cells, were cultured in the medium of DMEM/F12 supplemented by 10% FBS. All cultures were incubated at 37°C in humid conditions containing 5% CO_2_.

### Primary culture and identification of TCs in 2D mammary gland stromal cells culture

Isolated normal mammary gland derived stromal cells referring to Allinen *et al*. [36] with some modifications changing four cell strainers including 500 μ, 250 μ, 100 μ and 40 μ to two sieve of 250 μ and 40 μ. In brief, collected normal mammary gland from mid-pregnant BALB/c mice was minced to small fragments, digested with collagenase I (100 U/ml)/hyaluronidase (150 U/ml), diluted in DMEM/F12 medium without FBS, incubated in a mixture of tissue and digestion solutions at 37°C, then filtered through sieve mesh of different sizes, after centrifugation and suspended precipitation and was cultured in DMEM/F12 containing 10% FBS. We then characterized stromal cells through expression of vimentin, α-SMA, CK14, CK18 and Desmin [37]. To identify TCs, the primary culture cells were observed by phase contrast microscope.

### Supravital methylene blue staining

The supravital methylene staining according to the method mentioned in published papers [1, 6, 38] was used to identify TCs in primary mammary gland interstitial cells. In brief, phenol red-free DMEM was pre-warmed to wash cells culture, which was then incubated in 0.02% methylene blue solution at 37°C for 20 min. We quickly washed cells with phenol red-free DMEM medium, and observed results under Olympus microscope and acquired pictures as quickly as possible to avoid contamination.

### Mitochondria-specific labelling

TMRM (Immunochemistry Technologies, Bloomington, MN, USA) was used to label mitochondria specifically. Cells grown on cover slips were removed from culture dish and incubated in TMRM dissolved in PBS at 37°C for 15 min., then washed with PBS and label nucleus with Hoechst 33258. The results were observed with Zeiss confocal microscope (Zeiss 510 META; Zeiss, Oberkochen, Germany).

### Reconstituted 3D breast cancer tissue *in vitro*

Breast cancer tissues *in vitro* were reconstituted by mixing EMT-6 and normal mammary gland interstitial cells after three passages (1:1) with collagen I/Matrigel mixture as previously described [39]. In brief, 0.5 ml of concentrated 2X H-DMEM medium containing 10% FBS was mixed with 0.5 ml rat tail collagen and Matrigel in 4:1 (v/v),and then the mixture was neutralized quickly by 0.1 mol/l NaOH at low temperature, mixing 1.0 × 10^6^ EMT-6 and 1.0 × 10^6^ interstitial cells with scaffold and mixture was pipetted into casting moulds for incubation at 37°C. one millilitre DMEM/F12 containing 10% FBS was seeded to the dish after 60 min. of incubation, and the culture medium was changed daily. Cancer tissue sheet of EMT-6 alone was reconstituted following the same procedures with the number of 1 × 10^6^ as control group.

### Histology and immunohistological staining

Samples obtained at 3, 5, and 7 days were fixed in 4% formaldehyde and embedded in paraffin. Sections of 3 μm thickness were cut for haematoxylin and eosin staining as regular procedures. For immunohistochemistry, the primary antibodies used were: α-smooth muscle actin (diluted 1:800; Sigma-Aldrich), vimentin (diluted 1:800; Santa Cruz Biotechnology, Inc., CA, USA), c-kit/CD117 (diluted 1:200), E-cadherin (Abcam clone decma-1, dilution 1:800), collagen IV (diluted 1:200), pan-CK (diluted 1:200), PCNA (diluted 1:200). Sections were incubated with primary antibodies overnight at 4°C. Then, biotin-labelled secondary antibodies were used and finally detected with diaminbenzidine (Sigma-Aldrich). Nuclei were stained by haematoxylin. The number of PCNA-positive nuclei was estimated in 1000 randomly scored cells of each reconstituted tissue sheets and expressed in per cent as PCNA index.

### Immunofluorescence and confocal microscopy

For immunofluorescence, the primary antibodies were CD34 (diluted 1:100), CK14 (Santa Cruz Biotechnology, Inc., diluted 1:200), CK18 (diluted 1:100), Desmin (diluted 1:200), c-kit/CD117 (diluted 1:200), vimentin (diluted 1:800) and pan-CK (diluted 1:200). Sections of samples were incubated with primary antibodies overnight at 4°C, then were incubated with FITC-labelled goat anti-mouse IgG or FITC-labelled rabbit anti-rat IgG, Cy3-labelled goat anti-mouse IgG or Cy3-labelled goat anti-rabbit IgG as the secondary antibodies. Hoechst 33258 was used to stain the nucleus. The results were observed under a Zeiss confocal microscope (Zeiss 510 META; Zeiss, Oberkochen, Germany) with BioRad confocal software (Bio-Rad Laboratories, Inc., CA, USA). The expression of F-actin was detected by Phalloidin-FITC, and procedures used were the following: sections were deparaffinized and permeabilized with 0.1% Triton X-100 in PBS, then stained with 50 mg/ml fluorescent phalloidin conjugate solution in PBS (containing 1% DMSO from the original stock solution) for 40 min. at room temperature.

### Transmission electron microscopy (TEM)

The EMT-6/stromal cells reconstituted breast cancer tissue samples were fixed in 2.5% glutaraldehyde containing 0.1 mol/l sodium cacodylate buffer (pH 7.4) for 6 hrs, postfixed in 1% phosphate-buffered OsO_4_ (pH 7.4) and embedded in epoxy resin. Toluidine blue in 0.1 M borate buffer was used to stain semi-thin section. The sections were observed under a light microscope and cut ultra-thin sections were examined by TEM (Technai10; Philip, Eindhoven, Netherlands).

### TUNNEL Assays

Apoptosis was examined following the manual of MEBSTAIN Apoptosis kit II (MBL MED. &BIO. Co., Nagoya, Japan). In brief, sections were incubated with proteinase K solution for 30 min. at 37°C. TdT solution was pipetted to label DNA nick end of samples, and then Avidin-FITC II solution was used to preceed histochemistry which was followed by counterstaining with Propidium Iodide. Finally, the positive stained cells were observed and counted under microscopy. We randomly took five pictures per section under 20× fields and repeated the same procedure three times at least.

### Statistical analysis

At least three independent experiments were assessed. Data were evaluated for significance through Student *t* tests; ANOVA was used to compare the data of cells going through apoptosis. Data were considered statistically significant at *P* < 0.05.

## Results

### Identification of TCs in primary cultured cells derived from normal mammary gland

**Fig. 1:**
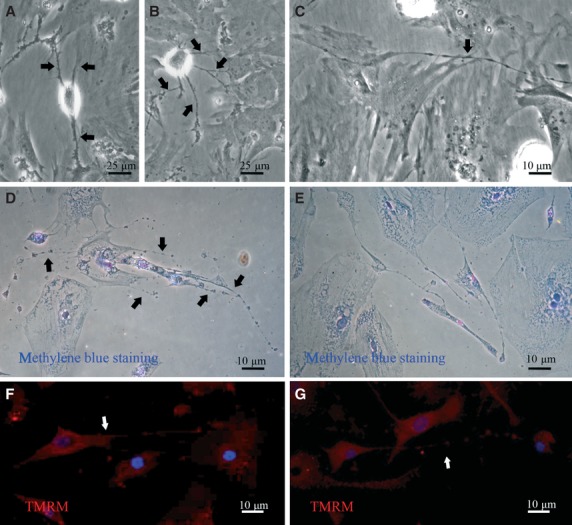
Telocytes in 2D breast stromal cells: phase contrast microscopy, supravital methylene blue staining and mitochondria-specific staining. (**A**) Fusiform TCs with long processes (indicated by arrow). (**B**) Polygonal TCs with four long, thin prolongations (indicated by arrows). (**C**) Triangular TCs with bead-like telopodes, eight dilations in telopodes were visible (indicated by arrows). (**D**) and (**E**) Supravital methylene blue staining of TCs. An octopus-like telocyte with five bead-like telopodes (indicated by arrows) in D. TCs connected with surrounding cells to form a network by their telopodes with bead-like conformation in E. (**F**) and (**G**) Mitochondria immunolabelled with TMRM of TCs. The strong prolongation with dilations of telocyte could be observed in primary breast stroma cell culture. The prolongations were indicated by arrows.

In 2D primary cultured mammary gland interstitial cells, we observed cells with suggestive morphology for TCs by phase contrast microscopy. These cells have long, slender prolongations extended from fusiform (Fig. 1A), polygonal (Fig. 1B) or triangular (Fig. 1C) cell body. The long and thin moniliform prolongations (Telopodes-Tps) suddenly emerge from the spindle cell body as is shown in Figure 1A. They are an alternation of thin segments-podomers-and dilated segments-podoms. TCs with polygonal cell body (indicated by arrows) shared at least four telopodes connecting with surrounding stromal cells (Fig. 1B). The numbers of the beads along the string of prolongations within TCs were quite different. A typical Tp is presented in Figure 1C having at least eight podoms (indicated by arrows).

The existence of TCs in 2D breast stromal cells monolayer culture was further confirmed by supravital methylene blue staining. An ‘octopus-like’ TC with at least five Tps could be observed in cell culture monolayer, clearly stained by methylene blue dye (Fig. 1D). TCs could form networks as it is shown in Figure 1E, the intricate interstitial networks could be constructed by telopodes and the cell body of TCs showed elongated appearance. Other vital staining, mitochondria-specific labelling with TMRM showed that TCs shared long and moniliform processes with dilations containing mitochondria (indicated by arrows; Fig. 1F and G).

**Fig. 2:**
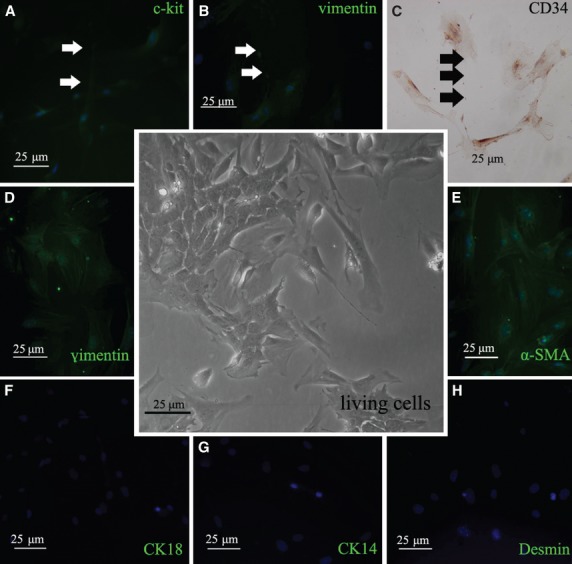
Immunofluorescence and immunohistochemistry of TCs and identification of mammary gland stromal cells in 2D culture. The central inset indicated stromal cells derived from breast tissue with different morphology of cells observed under phase contrast microscope. (**A**) Fluorescence microscopic observation showed c-kit/CD117-positive cells with obviously telopodes (green), and dilations were pointed by arrows. (**B**) Immunofluorescent staining result of vimentin-positive TCs with long prolongation (green, indicated by arrows). (**C**) Immunohistochemical staining result of CD34-positive TCs with telopodes. The dilations were indicated by arrows. (**D**–**H**) Immunofluorescent staining for other stromal cells in 2D monolayer cell culture. (D) vimentin-positive cells, (E) α-SMA-positive cells, (F) CK18-negative cells, (G) CK 14-negative cells, (H) Desmin-negative cells.

The common markers of TCs including c-kit/CD117 (Fig. 2A), vimentin (Fig. 2B) and CD34 (Fig. 2C) were detected in primary culture cells. In all the detected markers, podiums could be observed. These markers are mainly expressed within cytoplasm, including Tps, but with a discrete expression pattern. These results also confirmed the existence of TCs in 2D monolayer culture of breast stromal cells.

Meanwhile, we excluded the populations of epithelial cells in 2D monolayer cell culture by detecting the expression of stromal cells markers. The results showed that the cells were vimentin^+^ and α-SMA^+^ (Fig. 2D and E), and we could not detect or rarely detected the expression of CK18, CK14 and Desmin in cells, indicating that there were no epithelial cells in the stromal cells population (Fig. 2F–H). The stromal cells could be used to reconstitute breast cancer tissues *in vitro*.

### Immunohistochemical characterization of TCs and distribution of EMT-6 cells in EMT-6/stromal cells reconstituted breast cancer tissue *in vitro*

**Fig. 3:**
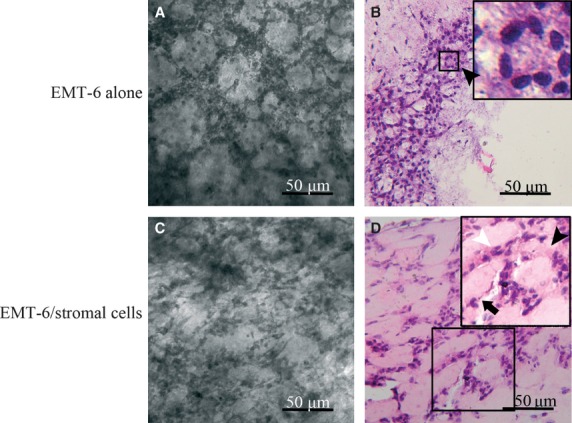
Comparison of self-assembly breast cancer structures between two groups of reconstituted breast cancer tissue sheets. (**A**) EMT-6 alone reconstituted breast cancer tissues were observed by phase contrast microscopy. (**B**) H&E staining of EMT-6 alone reconstituted breast cancer tissue at the seventh day of culture. Black arrowhead indicates nest structures. (**C**) EMT-6/stromal cells reconstituted breast cancer tissues were observed by phase contrast microscopy. (**D**) H&E staining of EMT-6/stromal cells reconstituted breast cancer tissue at the seventh day of culture. Black arrowhead indicates nest structures and white arrowhead indicates cord-like structures. Black arrow indicates stromal cells.

We constructed breast cancer tissues by combining EMT-6 with normal stromal cells derived from breast tissue *in vitro* by using collagen I/Matrigel scaffolds. Reconstituted breast cancer tissue constructed by EMT-6 alone was used as control. Cells mixed with collagen I/Matrigel scaffold contracted at day 1 after cultivation, and most of cells self-assembly to typical nest structure due to the static strength of glass tube (Fig. 3A–D). The black arrowheads indicated the nest structure formed in the reconstituted breast cancer tissue. During the culture, the morphological differences between the tissue sheets of EMT-6 alone and EMT-6/stromal reconstituted breast cancer tissue can be obviously observed under light microscope (Fig. 3A and C). In tissue sheets of EMT-6 cells alone, cells presented more aggregated alignment compressing to each other (Fig. 3A). Cells in the sheets of EMT-6/stromal cells reconstituted breast cancer tissue exhibited a spread-out appearance and well distributed well within the whole tissue (Fig. 3C). The differences of these two kinds of cancer tissue sheets could be compared by using haematoxylin and eosin staining. It can be observed obviously that some nest structures (indicated by black arrowhead) formed in these sheets, especially some cord-like structures (indicated by white arrowhead) in EMT-6/stromal cells reconstituted breast cancer tissue (Fig. 3D). In cancer tissue sheets of EMT-6 alone, the nest structures seemed more condensed and located mainly in the tissue edge (Fig. 3B). In contrast to this morphology, the nest structures exhibited a more homogenous appearance distributed in the whole EMT-6/stromal cells tissue sheets. Stromal cells dispersed separately and were often located around breast cancer cells (indicated by black arrow) (Fig. 3D).

**Fig. 4:**
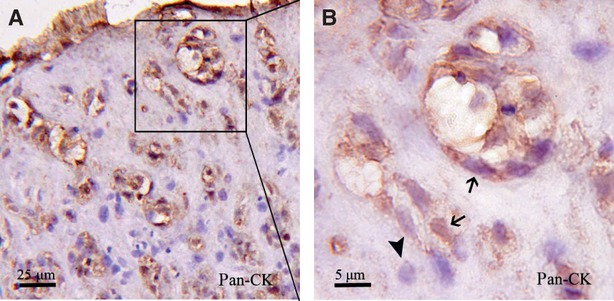
Immunohistochemical staining of pan-CK was detected in EMT-6/stromal cells reconstituted breast cancer. (**A**) pan-CK-positive EMT-6 cells dispersed in whole reconstituted breast cancer. (**B**) Higher magnification of the nest structures. EMT-6 (indicated by arrows) presented in reconstituted breast cancer tissue, and the stromal cells (indicated by arrowhead) accompanied by EMT-6 cells.

We then determined the distribution of EMT-6 by using immunohistochemistry of epithelia specific marker, pan-CK in EMT-6/stromal cells reconstituted breast cancer tissue (Fig. 4A and B). EMT-6 cells clustered and self-assembled to nest structures. pan-CK-positive EMT-6 (arrow in Fig. 4B) cells distributed in the whole breast cancer tissues sheets. Stromal cells (arrowhead in Fig. 4B) distributed adjacent to EMT-6 cells, suggesting the complexity of cell types in these models, which mimic the cellular composition of breast cancer tissue *in vivo*.

**Fig. 5:**
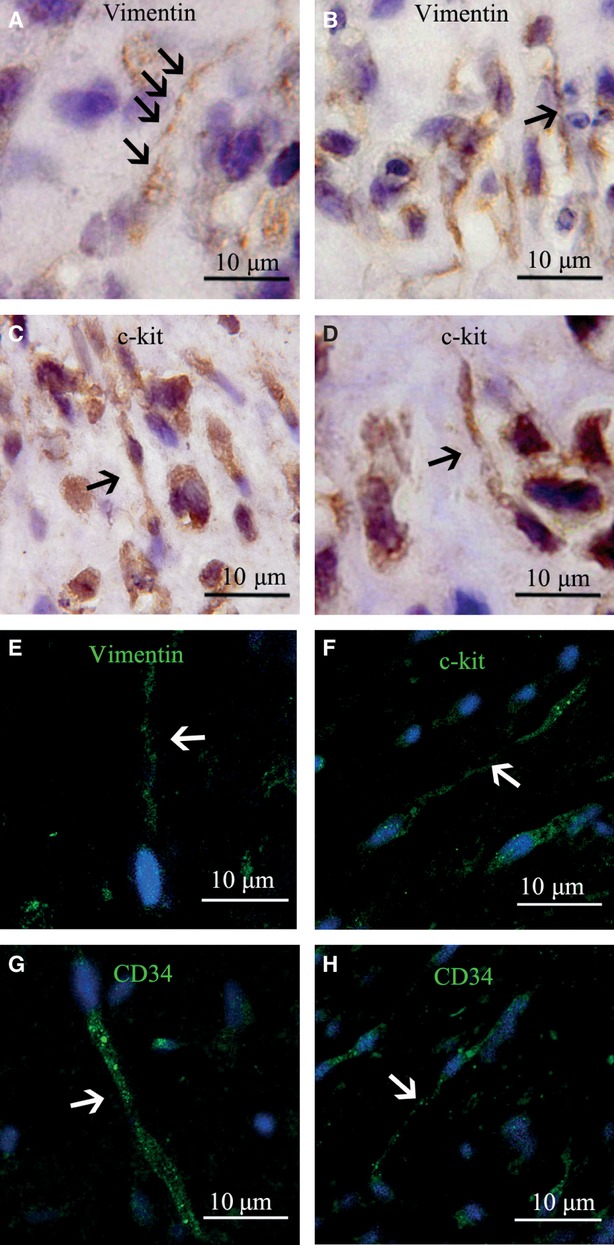
Immunohistochemical and immunofluorescent morphology of TCs in EMT-6/stromal cells reconstituted breast cancer tissue. (**A**–**D**) Immunohistochemical staining of TCs. (A) and (B) TCs were positive for vimentin with clear telopodes with dilations in A and with long prolongations in B (indicated by arrows, respectively). (C) and (D) TCs were positive for c-kit/CD117. (**E**–**H**) Immunofluorescent staining of TCs. The arrows indicate TCs. (E) Vimentin-positive TCs detected by immunofluorescence. (F) c-kit/CD117-positive TCs detected by immunofluorescence. (G) and (H) CD34-positive TCs detected by immunofluorescence.

TCs in EMT-6/stromal cells reconstituted breast cancer tissue were paid more attention. Although there are no specific markers only for TCs, c-kit/CD117, CD34 and vimentin can be used in combination to identify them. We performed immunohistochemistry and immunofluorescence staining to determine vimentin^+^, c-kit/CD117^+^ and CD34^+^ TCs in EMT-6/stromal cells reconstituted breast cancer tissue (Fig. 5). The arrows indicated that TCs with distinctive thin prolongations could accompany other cells, and vimentin was mainly located along the process of cells (Fig. 5A and B). In addition, we found that EMT6 breast cancer cells were c-kit/CD117-positive (Fig. 5C and D), which confirmed the observations reported by other group [40], but the distinct morphology made TCs clearly distinguished from other periphery breast cancer cells within reconstituted tumour tissue. Moreover, the immunofluorescent staining exhibited the expression of vimentin (Fig. 5E), c-kit/CD117 (Fig. 5F) and CD34 (Fig. 5G and H) in typical TCs, which distributed intermittently instead of continuously in the whole cell body.

### Spatial location of TCs and EMT-6 cells in EMT-6/stromal cells reconstituted breast cancer tissue

**Fig. 6:**
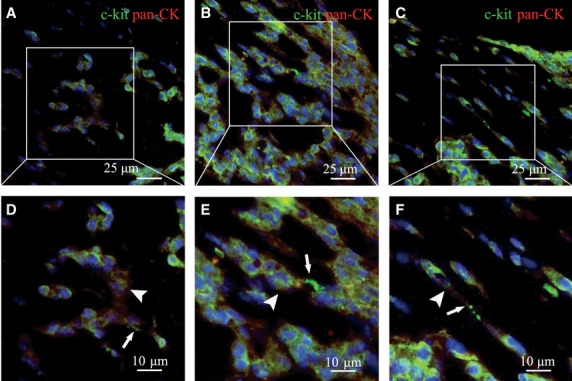
The spatial relationship between TCs and EMT-6 cells in EMT-6/stromal cells reconstituted breast cancer tissue was observed by double immunofluorescence staining. (**A**–**C**) c-kit/CD117^+^ cells was labelled with green colour and pan-CK^+^ cells was labelled with red colour. The nuclei were stained by Hoechst 33258. (**D**–**F**) Higher magnification of A, B and C. c-kit/CD117^+^ TCs distributed in the reconstituted breast cancer tissues (indicated by arrows), which are located close to pan-CK^+^ EMT-6 cells nest structure. EMT-6 cells were red stained and are indicated by arrowheads.

To investigate the potential roles of TCs in the organization of breast cancer tissues *in vitro*, we applied c-kit/CD117 and pan-CK double immunofluorescent staining to examine the spatial intercellular relationship between TCs and EMT-6 breast cancer cells in reconstituted tissue sheets. In Figure 6A and B, red fluorescence-stained pan-CK^+^ breast cancer cells self-assembled into nest structures, and green fluorescence-stained c-kit/CD117^+^ TCs were closely located around the cancer cells. Although some EMT6 cancer cells were with faint c-kit/CD117 staining, the pronounced green fluorescence and typical morphology of TCs could be identified in reconstituted cancer tissue sheets. The intercellular interaction between TCs and other cells exhibited different appearance. In Figure 6A and D, c-kit/CD117^+^ cells (indicated by white arrow) invaded into the clustered cell nest and contacted the proximal cancer cells (indicated by white arrowhead). Additionally, c-kit/CD117^+^ cells were situated at the niche of nest across the adjacent cancer cell clusters (Fig. 6B and E). The typical telopode features of c-kit/CD117^+^ TCs could be observed clearly in Figure 6C and F. The long process of TCs (indicated by white arrow) spread on the surface of the intercellular interaction area and was overlaid with the bottom dark red-stained pan-CK^+^ cytosol of breast cancer cells (indicated by white arrowhead). These results revealed that TCs could accompany, contact and link with EMT-6 cancer cells, which seemed to be participating in the self-assembly of nest structures of reconstituted breast cancer tissue.

**Fig. 7:**
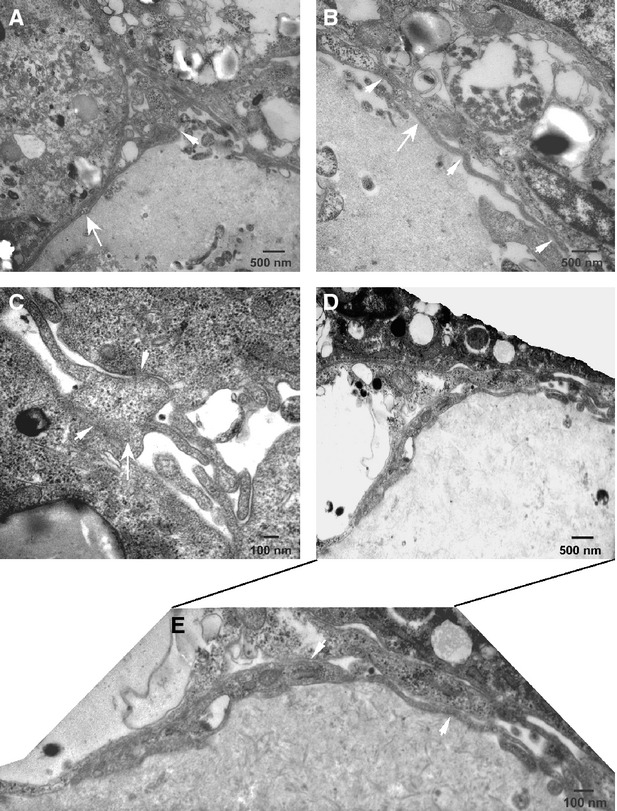
The transmission electron microscopy showed the interaction between telocyte-like cells and adjacent cells in EMT-6/stromal cells reconstituted breast cancer tissue. (**A**–**D**) The long and thin telopodes of telocyte-like cells (white arrow) exhibited distinct contact points (white arrow head) with the membrane of other cells. (**E**) Higher magnification of (D).

The TEM observation of EMT-6/stromal cells reconstituted breast cancer tissue sections showed that the cells (indicated by white arrow), which resembled the appearance of TCs with thin and long process extend along the intercellular space of EMT6 breast cancer cells and interstitial cells (Fig. 7A–D). The telocyte-like cells interacted with breast cancer cells and other stromal cells through membrane-to-membrane contact (indicated by white arrow head). In Figure 7B, it could be observed clearly that a telocyte-like cell exhibited a very long and tortuous telopodes with five corner protuberances communicating with adjacent cells (indicated by white arrow head). It also could be found to be embedded into a cluster of cells, which in close contact with the surrounding cells. Figure 7C showed a cross-section of a telocyte-like cell. It was located among three cells and might serve as a bridge that directly linked the adjacent cells with membrane-to-membrane contact. This kind of typical transcellular interaction could also be observed in Figure 7D and E. These bridging structures further suggested the potential roles of telocyte-like cells in the self-assembly of reconstituted breast cancer tissue.

### Potential function of TCs in self-assembly of EMT-6/stromal cells reconstituted breast cancer tissue

The different morphology between EMT-6/stromal cells and EMT-6 alone reconstituted breast cancer tissue, which has been described above, provided clues that the supplement of primary stromal cells containing TCs might have potential effects on the biological phenotypes of reconstituted breast cancer tissue. We subsequently compared the ability of cell proliferation and resistance to apoptosis of cancer cells in two tissue groups.

**Fig. 8:**
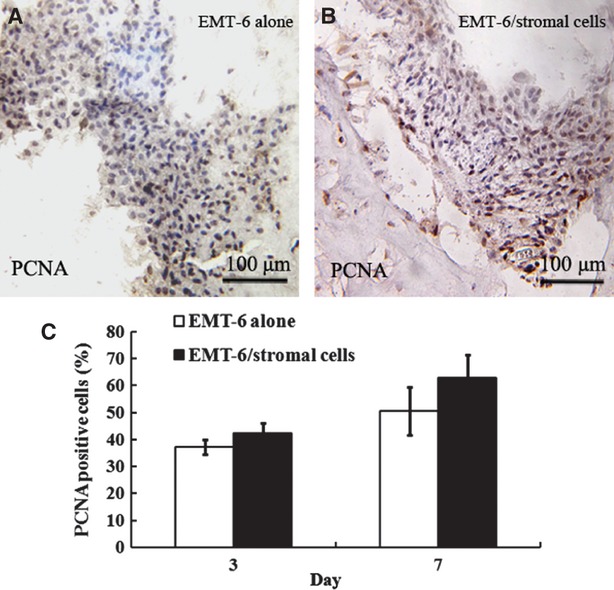
Comparison of the proliferation index by detecting the percentage of PCNA-positive cells in two groups of reconstituted breast cancer tissue. Immunohistochemical analysis of the expression of PCNA was evaluated in EMT-6 alone (**A**) and EMT-6/stromal cells reconstituted breast cancer tissue (**B**). Quantification of the proliferation index of PCNA-positive cells in EMT-6/stromal cells and EMT-6 alone reconstituted breast cancer tissue is shown in (**C**). Bars show the standard error of the mean.

As proliferation rate is an important factor determining the tumour aggressiveness, the evaluation of PCNA index (the percentage of PCNA- immunopositive nuclei in the investigated tissue sample) is suggested as useful in evaluating the malignancy of breast cancer. We then compared the percentage of PCNA-positive cells after 3 and 7 days culture in these two reconstituted breast cancer tissues (Fig. 8A and B). The proliferation index of cells in the EMT-6/stromal cells reconstituted tissue sheets in the 3 days' culture with the mean of 42.4%, which contrasts with 37.2% in tissue sheets of EMT-6 alone. After 7 days' culturing, the proliferation index in EMT-6/stromal cells tissue sheets reached 62.8% *versus* 50.53% in tissue sheets of EMT-6 alone (Fig. 8C). A higher proliferation index was acquired in EMT-6/stromal cells reconstituted breast cancer tissue than in the tissue sheets of EMT-6 alone. This result suggested that TCs and other stromal cells might improve cancer cell growth to some degree.

**Fig. 9:**
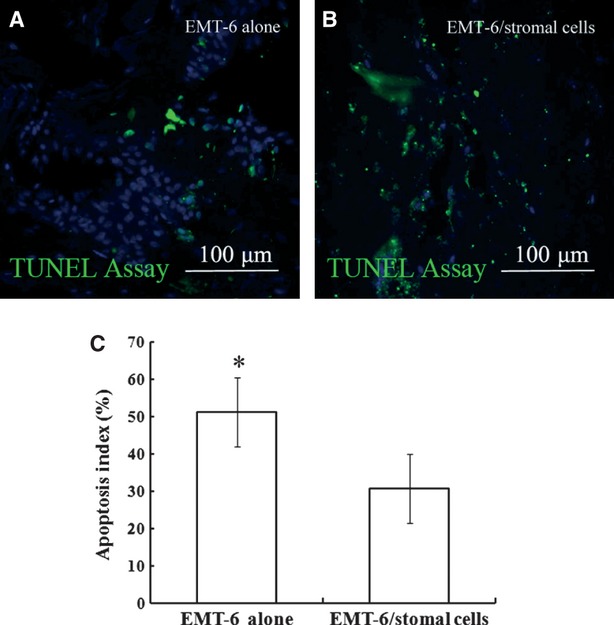
Apoptosis was detected by using TUNEL assay. Apoptosis index was measured by TUNEL assay in EMT-6 alone (**A**) and EMT-6/stromal cells reconstituted breast cancer tissue (**B**). Quantification of the apoptosis index of cells in EMT-6/stromal cells and EMT-6 alone reconstituted breast cancer tissues is shown in (**C**). At least three independent experiments were assessed. Bars show the standard error of the mean. Data were evaluated for significance through Student *t* tests; ANOVA was used to compare the data of cells going through apoptosis. Data were considered statistically significant at *P* < 0.05, which is indicated as *.

The characteristic features of breast cancer are the property of deregulated proliferation and acquired ability of resistance to apoptosis. Through quantitative comparison of the apoptotic rate between the two groups of reconstituted breast cancer tissue by using TUNEL assay, we found that the percentage of cells going through apoptosis in tissue sheets of EMT-6 alone was greater than that of cells in EMT-6/stromal cells reconstituted breast cancer tissue (Fig. 9). In other words, TCs and other stromal cells obviously attenuated apoptosis and prompted survival of EMT-6 breast cancer cells. The supplement of stromal cells containing TCs in EMT-6/stromal cells reconstituted breast cancer tissue could not only affect the self-assembly of cancer tissues but also influence the proliferation and apoptosis of breast cancer cells.

**Fig. 10:**
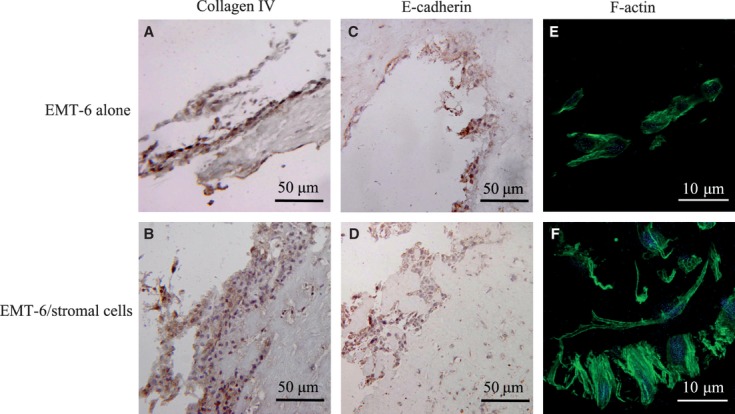
The expression of collagen IV, E-cadherin and F-actin in reconstituted breast cancer tissue was observed. (**A**) and (**B**) The expression of collagen IV was detected by immunohistochemistry in EMT-6 cells alone (A) and EMT-6/stromal cells (B) reconstituted breast cancer tissue respectively. (**C**) and (**D**) The expression of E-cadherin was detected by using immunohistochemistry in EMT-6 alone and EMT-6/stromal cells reconstituted breast cancer tissue, respectively. (**E**) and (**F**) Detection of F-actin by using immunofluorescence in EMT-6 alone and EMT-6/stromal cells reconstituted breast cancer tissue, respectively.

In addition, we evaluated the effect of stromal cells containing TCs on the organization of extracellular matrix, cell–cell junction and cytoskeleton assembly by comparison of the expression of collagen IV, E-cadherin and F-actin between these two groups of reconstituted breast cancer tissue. The presence of collagen IV in EMT-6 alone and EMT-6/stromal cells cancer tissue sheets indicated the formation of basement membrane (Fig. 10A and B). All typical nest structure formed in both of them can express a marker of cell–cell junction, E-cadherin (Fig. 10C and D). These results revealed that cancer cells embedded in collagen I/Matrigel could secrete components of basement membrane that resemble the behaviour of cancer cells *in vivo*. Through detection of F-actin microfilament by FITC-phalloidine, we found that F-actin is mainly located in the cytoplasm and the filaments seemed prominent at the interface of cell–cell contacts (Fig. 10E and F). The F-actin filament in tissue sheets of EMT-6 cells alone exhibited very short and slim filament without pronounced actin bundles (Fig. 10E). Contrast to that appearance, the F-actin filament in EMT-6/stromal cells reconstituted breast cancer tissue presented a well-assembled pattern with thick bundles, and aggregated along the boundary of intercellular contacted areas (Fig. 10F). As the F-actin filaments are the important component of cytoskeletons which closely relate to cell movements, the differences in F-actin assembly revealed the higher movement ability of cells in EMT-6/stromal cells reconstituted breast cancer tissue than those in the tissue sheets of EMT-6 cells alone.

## Discussion

This study provided evidence that stromal cells containing TCs might influence the self-assembly of reconstituted breast cancer tissue. In combination of the immunohistochemical analysis and the TEM observation, we could identify and characterize TCs in reconstituted breast cancer tissue and investigate their relationship with cancer cells in spatial locations. These results might provide clues for exploration of the potential functions of TCs in tissue organization *in vitro*.

Breast cancer cells cultured in 3D scaffold materials could develop into tumour tissues [41]. There are some observations indicating that addition of stromal cells could potentiate the reconstitution of breast cancer in 3D culture systems than cultivating cancer cells alone [42]. Breast cancer cells possess the ability of self-assembly to form tissue-specific structures [43].These lines of evidence suggested that the incorporation of stromal cells of mammary gland has essential roles in improving the self-assembly formation of breast cancer tissue. TCs are the important components of stromal cells, which have revealed latent functions in other organs. In normal mammary gland, the balance of epithelium/stroma was supposed to be affected by TCs, which was considered a critical stromal component of the interstitial morpho-functional unit [6]. However, the roles of TCs in 3D reconstruction of breast cancer tissues are still under investigation. Therefore, it is of significant importance for the immunohistochemical characterization and functional identification of mammary gland TCs during the development of breast cancer tissues within scaffold biomaterials. There is also an urgent need to understand the specific features of TCs in engineered breast cancer tissues by applying immunohistological analysis, which may lay the foundation of further research on TCs.

Through TEM, we observed the telocyte-like cells spreading along the cellular membrane of other adjacent cells and interacted with each other through membrane-to-membrane contact. However, there is a significant discrepancy between normal tissues *in vivo* and reconstituted tissues *in vitro*. Compared with cells *in vivo* or cultured under 2D conditions, cells in the materials exhibit a significant variation of biological features, which contribute to the discrepancy of ultrastructure. We suppose that the change in living microenvironment of TCs might contribute to their characteristic variance observed under TEM. Moreover, the physical-chemical property and other features of biomaterial will influence the cellular morphology of transplanted cells. Therefore, it is of great importance to put forward the investigation of the immunohistochemical characters and functional features of TCs so as to lay the foundation for the acquirement of specific TEM diagnostic results in reconstituted tissues.

Assembly into specific structure was the basis of cytological behaviours of cancer cells. TCs seemed to play a potential role in manipulating the biological properties of breast cancer. The current study suggested that TCs and other stromal cells in the reconstituted breast cancer tissue might promote the growth of cancer cells and help them resistant to apoptosis. As there are some lines of evidence regarding the intercellular communication functions of TCs through transmission of multi-vesicular bodies to deliver the signal molecules [4, 44] and through gap junctions [1, 45], this kind of communication might still exist in the self-assembled breast cancer tissues, which might contribute cancer cells adaptive to tumour microenvironment including hypoxia, low pH and acidosis. Considering the spatial relationship between TCs and cancer cells that we observed in the study, TCs might be involved in the programming of potentiating cell growth and against apoptosis of breast cancer.

In conclusion, TCs with long, thin prolongations were detected and connected with other stromal cells to form a complex interstitial network in 2D cultured breast stromal cells. In EMT-6/stromal cells reconstituted breast cancer tissue *in vitro*, TCs survived and accompanied by cancer cells to exert potential functions, such as assisting the self-assembly of cells, inhibition of apoptosis, facilitating growth of cancer cells. Exploration of the potential function of TCs would be helpful to understand the mechanism of breast cancer assembly and find possible therapeutic strategies targeting tumour stromal microenvironment.

## References

[1] Popescu LM, Faussone-Pellegrini MS (2010). Telocytes-a case of serendipity: the winding way from interstitial cells of Cajal (ICC), via interstitial Cajal-like cells (ICLC) to Telocytes. J Cell Mol Med.

[2] Popescu LM, Andrei F, Hinescu ME (2005). Snapshots of mammary gland interstitial cells: methylene-blue vital staining and c-kit immunopositivity. J Cell Mol Med.

[3] Radu E, Regalia T, Ceafalan L (2005). Cajal-type cells from human mammary gland stroma: phenotype characteristics in cell culture. J Cell Mol Med.

[4] Gherghiceanu M, Popescu LM (2005). Interstitial Cajal-like cells (ICLC) in human resting mammary gland stroma. Transmission electron microscope (TEM) identification. J Cell Mol Med.

[5] Gherghiceanu M, Popescu LM (2009). Human epicardium: ultrastructural ancestry of mesothelium and mesenchymal cells. J Cell Mol Med.

[6] Kostin S, Popescu LM (2009). A distinct type of cell in myocardium: interstitial Cajal-like cells (ICLCs). J Cell Mol Med.

[7] Gherghiceanu M, Manole CG, Popescu LM (2010). Telocytes in endocardium: electron microscope evidence. J Cell Mol Med.

[8] Popescu LM, Gherghiceanu M, Suciu LC (2011). Telocytes and putative stem cells in the lungs: electron microscopy, electron tomography, and laser scanning microscopy. Cell Tissue Res.

[9] Gherghiceanu M, Hinescu ME, Andrei F (2008). Interstitial Cajal-like cells (ICLC) in myocardial sleeves of human pulmonary veins. J Cell Mol Med.

[10] Hashitani H, Lang RJ (2010). Functions of ICC-like cells in the urinary tract and male genital organs. J Cell Mol Med.

[11] Popescu LM, Ciontea SM, Cretoiu D (2007). Interstitial Cajal-like cells in human uterus and fallopian tube. Ann N Y Acad Sci.

[12] Suciu L, Popescu LM, Gherghiceanu M (2010). Telocytes in human term placenta: morphology and phenotype. Cells Tissues Organs.

[13] Hinescu ME, Ardeleanu C, Gherghiceanu M (2007). Interstitial Cajal-like cells in human gallbladder. J Mol Histol.

[14] Rusu MC, Nicolescu MI, Jianu AM (2012). Esophageal telocytes and hybrid morphologies. Cell Biol Int.

[15] Nicolescu MI, Popescu LM (2012). Telocytes in the interstitium of human exocrine pancreas: ultrastructural evidence. Pancreas.

[16] Popescu BO, Gherghiceanu M, Kostin S (2012). Telocytes in meninges and choroid plexus. Neurosci Lett.

[17] Ceafalan L, Gherghiceanu M, Popescu LM (2012). Telocytes in human skin–are they involved in skin regeneration?. J Cell Mol Med.

[18] Popescu LM, Ciontea SM, Cretoiu D (2005). Novel type of interstitial cell (Cajal-like) in human fallopian tub*e*. J Cell Mol Med.

[19] Manole CG, Cismasiu V, Gherghiceanu M (2011). Experimental acute myocardial infarction: telocytes involvement in neo-angiogenesis. J Cell Mol Med.

[20] Ardeleanu C, Bussolati G (2011). Telocytes are the common cell of origin of both PEComas and GISTs: an evidence-supported hypothesis. J Cell Mol Med.

[21] Kim JB, Stein R, O'Hare MJ (2012). A mechanism underlying the effects of polyunsaturated fatty acids on breast cancer. Int J Mol Med.

[22] Orimo A, Gupta PB, Sgroi DC (2005). Stromal fibroblasts present in invasive human breast carcinomas promote tumor growth and angiogenesis through elevated SDF-1/CXCL12 secretion. Cell.

[23] Mazzone M, Dettori D, Olivera RLD (2009). Heterozygous deficiency of PHD2 restores tumor oxygenation and inhibits metastasis via endothelial normalization. Cell.

[24] Vona-Davis L, Howard-McNatt M, Rose DP (2007). Adiposity, type 2 diabetes and the metabolic syndrome in breast cancer. Obes Rev.

[25] Vona-Davis L, Rose DP (2007). Adipokines as endocrine, paracrine, and autocrine factors in breast cancer risk and progression. Endocr Relat Cancer.

[26] Li LY, Li Y (2011). Optimizing a 3D culture system to study the interaction between epithelial breast cancer and its surrounding fibroblasts. J Cancer.

[27] Maffini MV, Calabro JM, Soto AM (2005). Stromal regulation of neoplastic development: age-dependent normalization of neoplastic mammary cells by mammary stroma. Am J Pathol.

[28] DeCosse JJ, Gossens CL, Kuzma JF (1973). Breast cancer: induction of differentiation by embryonic tissue. Science.

[29] Baker EL, Srivastava J, Yu DH (2011). Cancer cell migration: integrated roles of matrix mechanics and transforming potential. PLoS ONE.

[30] Viloria-Petit AM, David L, Jia JY (2009). A role for the TGFβ-Par6 polarity pathway in breast cancer progression. Proc Natl Acad Sci USA.

[31] Fischbach C, Kong HJ, Hsiong SX (2009). Cancer cell angiogenic capability is regulated by 3D culture and integrin engagement. Proc Natl Acad Sci USA.

[32] Olsen CJ, Moreira J, Lukanidin EM (2010). Human mammary fibroblasts stimulate invasion of breast cancer cells in a three-dimensional culture and increase stroma development in mouse xenografts. BMC Cancer.

[33] Sadlonova A, Novak Z, Johnson MR (2005). Breast fibroblasts modulate epithelial cell proliferation in three-dimensional *in vitro* co-culture. Breast Cancer Res.

[34] Manabe Y, Toda S, Miyazaki K (2003). Mature adipocytes, but not preadipocytes, promote the growth of breast carcinoma cells in collagen gel matrix culture through cancer-stromal cell interaction. J Pathol.

[35] Sigurdsson V, Hilmarsdottir B, Sigmundsdottir H (2011). Endothelial induced EMT in breast epithelial cells with stem cell properties. PLoS ONE.

[36] Allinen M, Beroukhim R, Cai L (2004). Molecular characterization of the tumor microenvironment in breast cancer. Cancer Cell.

[37] Santner SJ, Pauley RJ, Tait L (1997). Aromatase activity and expression in breast cancer and benign breast tissue stromal cells. J Clin Endocr Metab.

[38] Hinescu ME, Popescu LM (2005). Interstitial Cajallike cells (ICLC) in human atrial myocardium. J Cell Mol Med.

[39] Zhao YS, Wang CY, Li DX (2005). Construction of a unidirectionally beating 3-dimensional cardiac muscle construct. J Heart Lung Transplant.

[40] Edling CE, Hallberg B (2005). c-kit/CD117 a hematopoietic cell essential receptor tyrosine kinase. Int J Biochem Cell Biol.

[41] Becker JL, Blanchard DK (2007). Characterization of primary breast carcinomas grown in three-dimensional cultures. J Surg Res.

[42] Heneweer M (2005). Muusse, Dingemans M, *et al*. Co-culture of primary human mammary fibroblasts and MCF-7 cells as an *in vitro* breast cancer model. Toxicol Sci.

[43] Han J, Chang H, Giricz O (2010). Molecular predictors of 3D morphogenesis by breast cancer cell lines in 3D culture. PLoS Comput Biol.

[44] Bani D, Formigli L, Gherghiceanu M (2010). Telocytes as supporting cells for myocardial tissue organization in developing and adult heart. J Cell Mol Med.

[45] Mandache E, Popescu LM, Gherghiceanu M (2007). Myocardial interstitial Cajal-like cells (ICLC) and their nanostructural relationships with intercalated discs: shed vesicles as intermediates. J Cell Mol Med.

